# Regulation of human cardiac potassium channels by full-length KCNE3 and KCNE4

**DOI:** 10.1038/srep38412

**Published:** 2016-12-06

**Authors:** Geoffrey W. Abbott

**Affiliations:** 1 Bioelectricity Laboratory, Dept. of Pharmacology and Dept. of Physiology and Biophysics, School of Medicine, University of California, Irvine, CA, USA

## Abstract

Voltage-gated potassium (Kv) channels comprise pore-forming α subunits and a multiplicity of regulatory proteins, including the cardiac-expressed and cardiac arrhythmia-linked transmembrane KCNE subunits. After recently uncovering novel, N-terminally extended (L) KCNE3 and KCNE4 isoforms and detecting their transcripts in human atrium, reported here are their functional effects on human cardiac Kv channel α subunits expressed in *Xenopus laevis* oocytes. As previously reported for short isoforms KCNE3S and KCNE4S, KCNE3L inhibited hERG; KCNE4L inhibited Kv1.1; neither form regulated the HCN1 pacemaker channel. Unlike KCNE4S, KCNE4L was a potent inhibitor of Kv4.2 and Kv4.3; co-expression of cytosolic β subunit KChIP2, which regulates Kv4 channels in cardiac myocytes, partially relieved Kv4.3 but not Kv4.2 inhibition. Inhibition of Kv4.2 and Kv4.3 by KCNE3L was weaker, and its inhibition of Kv4.2 abolished by KChIP2. KCNE3L and KCNE4L also exhibited subunit-specific effects on Kv4 channel complex inactivation kinetics, voltage dependence and recovery. Further supporting the potential physiological significance of the robust functional effects of KCNE4L on Kv4 channels, KCNE4L protein was detected in human atrium, where it co-localized with Kv4.3. The findings establish functional effects of novel human cardiac-expressed KCNE isoforms and further contribute to our understanding of the potential mechanisms influencing cardiomyocyte repolarization.

There are forty known Voltage-gated potassium (Kv) channel α subunit genes in the human genome, each of which encodes a functionally distinct pore-forming α subunit, contributing to the wide variety of native Kv currents observed *in vivo*. In addition, native Kv channels are large macromolecular complexes composed of multiple different pore-forming α subunits, regulatory (β) subunits, and other even more promiscuous regulatory proteins not limited to ion channel regulation. The various regulatory subunits further enhance the native Kv current repertoire, facilitate specialization for specific α subunits to conduct diverse functions in different cell types, and help to shape currents during different stages of development, or diversify Kv currents between sexes and different life stages by responding to hormones^1^,^2^,^3^.

The KCNE family of Kv β subunits, also known as MinK related peptides (MiRPs), comprises five subunits, each of which possesses a single transmembrane domain. Each of the KCNE proteins can regulate multiple different Kv channel α subunits^4^. This regulation can occur together with the presence of other regulatory subunits, and in some cases may be required for regulation by other types of regulatory subunit^5^,^6^. KCNE proteins are highly influential in the context of native potassium currents, in many cases altering fundamental channel properties including Voltage dependence^7^, gating kinetics^8^, and polarized trafficking^9^.

The crucial nature of KCNE proteins in many native channel complexes is highlighted by the detrimental and widespread consequences of *Kcne* gene deletion in mice^10^,^11^,^12^,^13^,^14^,^15^,^16^,^17^,^18^,^19^,^20^,^21^,^22^,^23^, and by the association of human *KCNE* gene sequence variants with disease states^24^,^25^,^26^,^27^,^28^. For example, variants in each of the human *KCNE* genes are either directly associated with potentially lethal cardiac arrhythmias or may influence arrhythmia predisposition in the presence of other environmental or genetic factors^29^,^30^. Kv channels are important for repolarization of cardiac myocytes, a process that ends each heart-beat in preparation for the next action potential and myocardial contraction. When sequence variants in cardiac Kv α or β subunits diminish function of the major repolarizing currents (*I*_Kr_ and *I*_Ks_) (generated by the hERG and KCNQ1 α subunits, with various β subunits), in the ventricles of the heart, this delays ventricular myocyte repolarization, extending the QT interval on the surface electrocardiogram (termed Long QT syndrome, LQTS). LQTS can predispose to perturbation of cardiac rhythm to the extent that the pumping function of the heart is disrupted, via a chaotic state termed ventricular fibrillation, which is often lethal^31^,^32^. Another potentially lethal cardiac arrhythmia syndrome, Brugada syndrome (BrS), is most commonly genetically linked to loss-of-function mutations in the *SCN5A* gene that encodes the primary cardiac sodium channel α subunits, Nav1.5^33^. However, more recently, sequence variants in the genes encoding subunits or putative subunits of cardiac channel complexes generating the transient outward Kv current (*I*_to_) have been associated with BrS. These include the genes encoding KCNE3 and KCNE5, which can regulate the Kv4.3 α subunit that generates *I*_to_ in human heart, and the *KCND3* gene that encodes Kv4.3 itself 34,35,36,37,38.

Given the necessity of KCNE subunits in some native channel complexes, the potential severity of disease states associated with some *KCNE* gene variants, and the possibility of remodeling of KCNE genes during disease states^39^, it is important to understand the molecular mechanisms underlying KCNE regulation of native Kv currents. Recently, I discovered exon 1 coding regions that generate longer (L) isoforms of human KCNE3 and KCNE4, detected both proteins in various epithelial tissues, and conducted an initial examination of their functional effects^40^. Here, I demonstrate the expression of KCNE4L protein in human heart, and describe the functional effects of KCNE3L and KCNE4L on the major human cardiac Kv channel α subunits.

## Materials and Methods

### Western blotting

Human atrial tissue was kindly provided by Dr. David Christini, Weill-Cornell Medical College (protocol #1206012437, approved by the Weill-Cornell Medical College Institutional Review Board (IRB)). Samples were handled in accordance with Weill-Cornell IRB guidelines and regulations, and informed consent was obtained from all subjects. Frozen atrial tissue samples were homogenized on dry ice using a mortar and pestle and resuspended in “MiRP buffer”: 150 mM NaCl, 50 mM Tris-HCL (pH 7.4), 20 mM NaF, 10 mM NaVO_4_, 1 mM phenylmethylsulfonyl fluoride (Thermo Fisher Scientific, Waltham, MA), 1% Nonidet P-40 (Pierce), 1% CHAPS (Sigma, St. Louis, MO, USA), 1% Triton X-100 (Thermo Fisher Scientific), and 1% SDS (Sigma) with protease inhibitor cocktail (Sigma). The suspension was rotated end-over-end at 4 °C for 4 hours, then centrifuged for 10 minutes at 3 × g at 4 °C. Supernatants were resuspended in LDS gel-loading buffer (Thermo Fisher) containing 25 mM tris(2-carboxyethyl)phosphine, heated for 10 minutes at 65 °C, vortexed, centrifuged for 3 minutes at 5 × g, and then separated by SDS-PAGE. Proteins were transferred (1 hour at 100 V) to PVDF membranes (BioRad, Hercules, CA, USA). After transfer, PVDF membranes were blocked in Tris-buffered saline (pH 7.6) containing 0.1% Tween-20 (TBST) and 5% dried milk for 1 h at room temperature, washed 3 × 5 minutes in TBST, and then incubated overnight at 4 °C with rabbit polyclonal primary antibodies in TBST containing 5% (w/v) dried milk. α-KCNE4L, a custom antibody (Genscript) raised to a peptide based on the KCNE4L-specific N-terminal segment (residues 17–32), sequence RAQSRTEQKNPLGLD-C (1/1000 dilutions)^40^ was used to probe for KCNE4L. Blots were next washed in TBST (3 × 5 minutes), incubated for 1 hour at room temperature with 1/5000 Horseradish peroxidase (HRP)-conjugated goat anti-rabbit IgG secondary antibody (Bio-Rad) in TBST containing 1% (w/v) dried milk, and then washed again in TBST (3 × 5 minutes) before chemiluminescent detection using Luminata Forte chemiluminescence substrate (Millipore, Temecula, CA), and analysis using a Gbox system and Gbox software (Syngene).

For Chinese hamster ovary (CHO) cell biochemistry, CHO cells were transfected with cDNAs encoding human KCNE4S in pCINeo, or human KCNE4L in pCDNA3.1+^40^. Two days post-transfection, cells were lysed in MiRP buffer, rotated end-over-end in Eppendorf tubes for 2 hours at 4 °C, then centrifuged (4000 × *g*; 10 min; 4 °C), and the resulting supernatants SDS-PAGE separated, western blotted and analyzed as described for heart samples above.

### Immunofluorescence detection

Human normal atrial tissue slides (5 μm thick, paraformaldehyde fixed and paraffin wax embedded) (2 per antibody combination) were purchased from Abcam (Cambridge, UK). Ventricular tissue samples from adult male mice (two per genotype) were similarly fixed, embedded and sectioned. Mice were housed and used according to the US National Institutes of Health *Guide for the Care and Use of Laboratory Animals.* Animal procedures were approved by the Animal Care and Use Committee at University of California, Irvine (IACUC Protocol #2011–2999). Human and mouse tissue sections were next deparaffinized and permeabilized, then immunofluorescence labeling was performed manually, with 3 × 5 minute washes in PBS between antibody incubation steps. Slides were incubated overnight at 4 °C with rabbit α-KCNE4L (in-house), goat α-KCNQ1 (Santa Cruz Biotechnology, Santa Cruz, CA), or mouse α-Kv4.3 (Neuromabs, Davis, CA) at 1/100 dilution in PBS with 1% BSA, followed by a 2 hour room-temperature incubation in Alexa-fluor-conjugated secondary antibodies (ThermoFisher Scientific, Waltham, MA) at 1/500 dilution in PBS. Following mounting using DAPI-containing slow-fade mounting medium (Life Technologies, Grand island, NY, USA), slides were viewed with an Olympus BX51 microscope and pictures were acquired using CellSens software (Olympus).

### Xenopus laevis oocyte expression

cRNA transcripts encoding hKCNE3L and hKCNE4L were generated by *in vitro* transcription (T7 polymerase mMessage mMachine kit, Thermo Fisher Scientific) from synthetic genes sub-cloned into pCDNA3.1+ with *Xenopus laevis* β-globin 5′ and 3′ UTRs flanking the coding region to enhance translation and cRNA stability, after vector linearization. hKCNE3S and hKCNE4S cRNAs were similarly generated, after using site-directed mutagenesis (Agilent, Santa Clara, CA), to disrupt the exon 1 start site on the cDNAs of their long counterparts to facilitate direct comparisons of short versus long isoforms in otherwise identical constructs. Human Kv4.2, Kv4.3 (full-length) and KChIP2 were similarly transcribed from cDNA templates also incorporating *Xenopus laevis* β-globin 5′ and 3′ UTRs (a kind gift of Dr. Steve A. N. Goldstein), as were human HCN1, hERG and Kv1.1. cRNA was quantified by spectrophotometry. Defolliculated stage V and VI *Xenopus laevis* oocytes (Ecocyte Bioscience, Austin, TX) were injected with one, two or three of the subunit cRNAs (5 ng of KCNE subunits, 4 ng hERG, 10 ng HCN1, 1–5 ng KChIP2, 0.1 ng Kv1.1, 1 ng Kv4.2, 1–5 ng Kv4.3 per oocyte). Oocytes were incubated at 16 °C in SBB solution (Ecocyte) containing penicillin and streptomycin, with daily washing, for 2–3 days before two-electrode voltage-clamp (TEVC) recording.

### TEVC

TEVC recording was performed at room temperature with an OC-725C amplifier (Warner Instruments, Hamden, CT) and pClamp8 software (Molecular Devices, Sunnyvale, CA). Oocytes were placed in a small-volume oocyte bath (Warner) and viewed with a dissection microscope. Bath solution was (in mM): 96 NaCl, 4 KCl, 1 MgCl_2_, 1 CaCl_2,_ 10 HEPES (pH 7.6); bath chemicals were from Sigma. TEVC pipettes were of 1–3 MΩ resistance when filled with 3 M KCl. Currents were recorded in response to a Voltage protocol consisting of pulses between −120 mV or −80 mV and 40 or 60 mV at 20 mV intervals, from a holding potential of −80 mV, to yield current-Voltage relationships and for fitting of inactivation kinetics. For hERG and Kv1.1, plotting peak current during a tail pulse to −30 mV, versus prepulse Voltage, permitted calculation of conductance-Voltage relationships. For quantification of Kv4.x steady-state inactivation, oocytes were held at −100 mV and prepulsed to Voltages between −120 and 0 mV followed by a tail pulse to +40 mV. For quantification of inactivation recovery rates, Kv4.x-based channels were double-pulsed to +40 mV with variable recovery times (10–5000 ms) at −120 mV in between, and the magnitude of the second peak compared to that of the initial peak for each pair. TEVC data analysis was performed with Clampfit (Molecular Devices) and Origin 6.1 (OriginLab Corp., Northampton, MA) software. Values are stated as mean ± SEM. Normalized tail currents (Kv4.x steady-state inactivation; hERG and Kv1.1 G/V relationships) were plotted versus prepulse voltage and fitted with a single Boltzmann function according to:


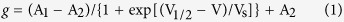


where *g* is the normalized tail conductance, A_1_ is the initial value at −∞, A_2_ is the final value at +∞, V_1/2_ is the half-maximal voltage of activation and V_s_ the slope factor. Kv4.x current inactivation curves were fitted with a standard (zero-shift) double exponential decay function with Chebyshev 4-point smoothing filter, whereas decay of Kv4.x-KChIP2 currents was amply described by a single exponential decay function. Inactivation recovery kinetics were fitted from mean normalized fractional recovery currents to a two-phase exponential association equation:


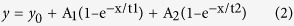


and in cases where iterative fitting yielded identical τ values, a single exponential fit was reported. Because mean inactivation recovery curves were fitted, these data are reported as a value with no standard error, but rather a chi-squared test for goodness of fit. In all other cases, values are reported with standard error of the mean. Where informative, currents were compared with one another using one-way ANOVA to assess statistical significance (P < 0.05). If multiple comparisons were performed, a post-hoc Tukey’s HSD test was performed following ANOVA.

## Results and Discussion

### Detection of KCNE4L protein in human atrium

**Figure 1:**
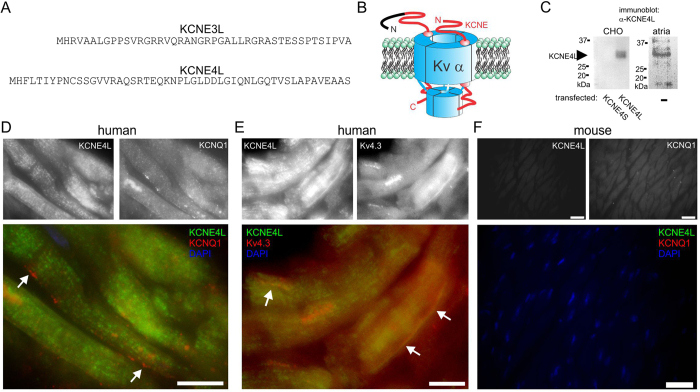
Full-length human KCNE4 protein is expressed in human atrium. (**A**) Predicted protein sequence for additional N-terminal residues of human KCNE3L (upper) and KCNE4L (lower) that are lacking in KCNE3S and KCNE4S. (**B**) Cartoon showing placement of N-terminal sequences from panel A at the N-terminal, extracellular end of the KCNE subunits (black portion). (**C**) Western blots probing expression of KCNE4L using an antibody raised to a peptide sequence specific to the long form of KCNE4. Bands were detected in lysates from human atria, and from CHO cells expressing KCNE4L, but not in those from CHO cells expressing KCNE4S. (**D**) Immunofluorescence images showing KCNE4L and KCNQ1 expression in human atrial myocytes. Upper, single-channel images; lower, merged images. Scale bar, 5 μm. Arrows, KCNQ1 with no co-localized KCNE4L. (**E**) Immunofluorescence images showing KCNE4L and Kv4.3 expression in human atrial myocytes. Upper, single-channel images; lower, merged images. Scale bar, 5 μm. Arrows, Kv4.3 co-localized with KCNE4L. (**F**) Immunofluorescence images showing negligible detection of KCNE4L and KCNQ1 in mouse ventricular myocytes. Upper, single-channel images; lower, merged images. Scale bar, 10 μm.

The novel exon 1-encoded N-terminal additions to human KCNE3 and KCNE4 extend their predicted coding regions by 44 and 51 residues, respectively, yielding predicted full-length proteins of 147 (KCNE3L) and 221 (KCNE4L) (Fig. 1A,B). In support of their potential relevance to human cardiac function, transcripts coding for KCNE3L and KCNE4L were previously detected in human atrial tissue^40^.

The reported migration patterns of the two forms of mature KCNE4 protein on SDS-PAGE gels correspond to ~25 kDa for KCNE4S^41^ and ~30 kDa for KCNE4L^40^. Here, an antibody raised to a peptide sequence present on only the long form of KCNE4 detected a ~30 kDa band in CHO cells expressing KCNE4L, but no bands in the 20–40 kDa size range in CHO cells expressing KCNE4S. Using aliquots of the same antibody stocks, a band at ~30 kDa (which corresponds to KCNE4L) was detected in homogenized human atrial tissue lysate (Fig. 1C). Thus, KCNE4L protein is expressed in human atrium. Similar studies were attempted for KCNE3, but did not yield unequivocal expression data for either KCNE3S or KCNE3L, and so are not included here.

Immunofluorescence staining of human atrial tissue with subunit-specific antibodies detected KCNE4L in a striated pattern in atrial myocytes, showing minimal overlap with KCNQ1 (Fig. 1D) but co-localization with Kv4.3 (Fig. 1E). As a negative control, mouse ventricular myocytes (which do not express KCNE4L and exhibit little evidence of KCNQ1 activity) were similarly probed, showing negligible signal, as expected if the antibodies are specific (Fig. 1F).

### KCNE3L inhibits hERG; KCNE4L inhibits Kv1.1; neither affects HCN1

**Figure 2:**
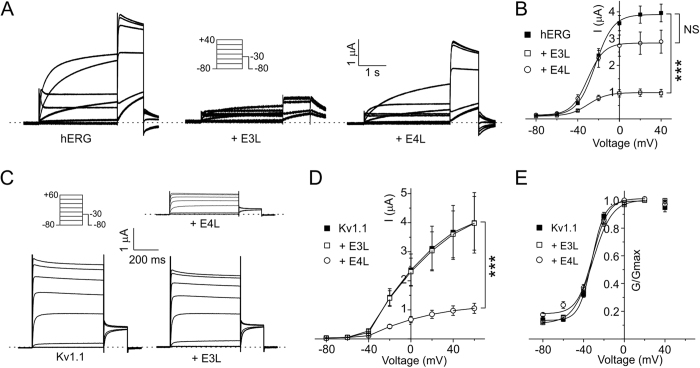
Functional effects of KCNE3L and KCNE4L on hERG, and human Kv1.1. (**A**) Exemplar current traces recorded from *Xenopus* oocytes expressing hERG alone (*n* = 25) or with KCNE3L (E3L; *n* = 18), or KCNE4L (E4L; *n* = 10). *Insets*: left, Voltage clamp protocol; right, scale bars. Zero current level indicated by dashed line. (**B**) Mean ± SEM peak current/voltage relationship for currents as in panel A; *n* values as in panel A. ***P < 1 × 10^−8^. NS, P > 0.05 (**C**) Exemplar current traces recorded from *Xenopus* oocytes expressing Kv1.1 alone (*n* = 26) or with KCNE3L (E3L; *n* = 19), or KCNE4L (E4L; *n* = 30). *Insets*: left, Voltage clamp protocol; right, scale bars. Zero current level indicated by dashed lines. (**D**) Mean ± SEM peak current/voltage relationship for currents as in panel C; *n* values as in panel C. ***P < 0.005. (**E**) Mean ± SEM normalized G/V relationship measured at beginning of tail pulse for currents as in panel C; *n* values as in panel C.

**Figure 3:**
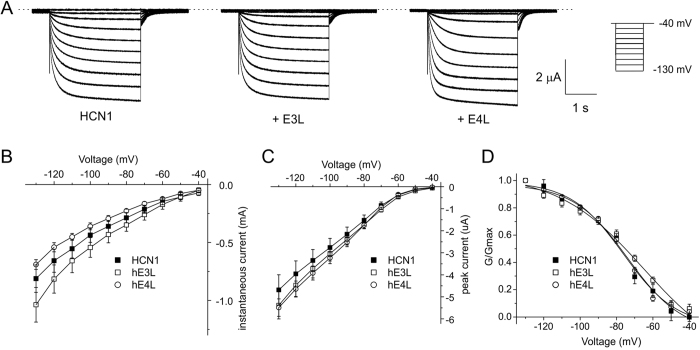
Absence of functional effects of KCNE3L and KCNE4L on human HCN1. (**A**) Exemplar current traces recorded from *Xenopus* oocytes expressing HCN1 alone (*n* = 13) or with KCNE3L (E3L; *n* = 17), or KCNE4L (E4L; *n* = 15). *Insets*: left, scale bars; right, Voltage clamp protocol. Zero current level indicated by dashed line. (**B**) Mean ± SEM instantaneous current/voltage relationship for currents as in panel A; *n* values as in (**A**). (**C**) Mean ± SEM peak current/voltage relationship for currents as in panel A; *n* values as in panel A. (**D**) Mean ± SEM normalized G/V relationship measured at beginning of tail pulse for currents as in panel A; *n* values as in panel A.

I recently found that KCNE3L converts KCNQ1 to a constitutively active K^+^ channel, as previously reported for KCNE3S^7^, although KCNE3L also diminished overall KCNQ1 current, in contrast to effects observed with KCNE3L^40^. KCNE3S inhibits KCNQ4 activity when expressed in *Xenopus* oocytes, whereas KCNE3L had no effect in similar experiments^7^,^40^. Previous studies also showed that in oocytes and in CHO cells, KCNE3S inhibits hERG^7^,^42^, which generates the major human cardiac repolarization current, *I*_Kr_^43^. Here, co-expression of hERG with KCNE3L in *Xenopus* oocytes recapitulated these findings, suggesting the extra N-terminal portion of KCNE3L does not substantively alter its interaction with hERG. As previously reported for KCNE4S^44^, KCNE4L did not alter hERG current (Fig. 2A,B). Kv1.1 was recently reported to be expressed in human and mouse heart and to influence susceptibility to atrial fibrillation^45^. Here, KCNE4L inhibited hKv1.1 activity, as previously found for KCNE4S^46^, whereas KCNE3L had no effect (Fig. 2C,D). The current remaining after co-expression of hKv1.1 with KCNE4L had similar Voltage dependence to that of homomeric hKv1.1 (Fig. 2E). KCNE2 is known to modulate the hyperpolarization-activated, monovalent cation-nonselective “pacemaker” channel encoded by HCN1^47^; here, neither KCNE3L nor KCNE4L exerted any functional effects when co-expressed with human HCN1, changing neither its instantaneous nor peak current magnitudes, nor its voltage dependence of activation (Fig. 3A–D).

### KCNE4L is a potent inhibitor of hKv4.2 regardless of KChIP2

**Figure 4:**
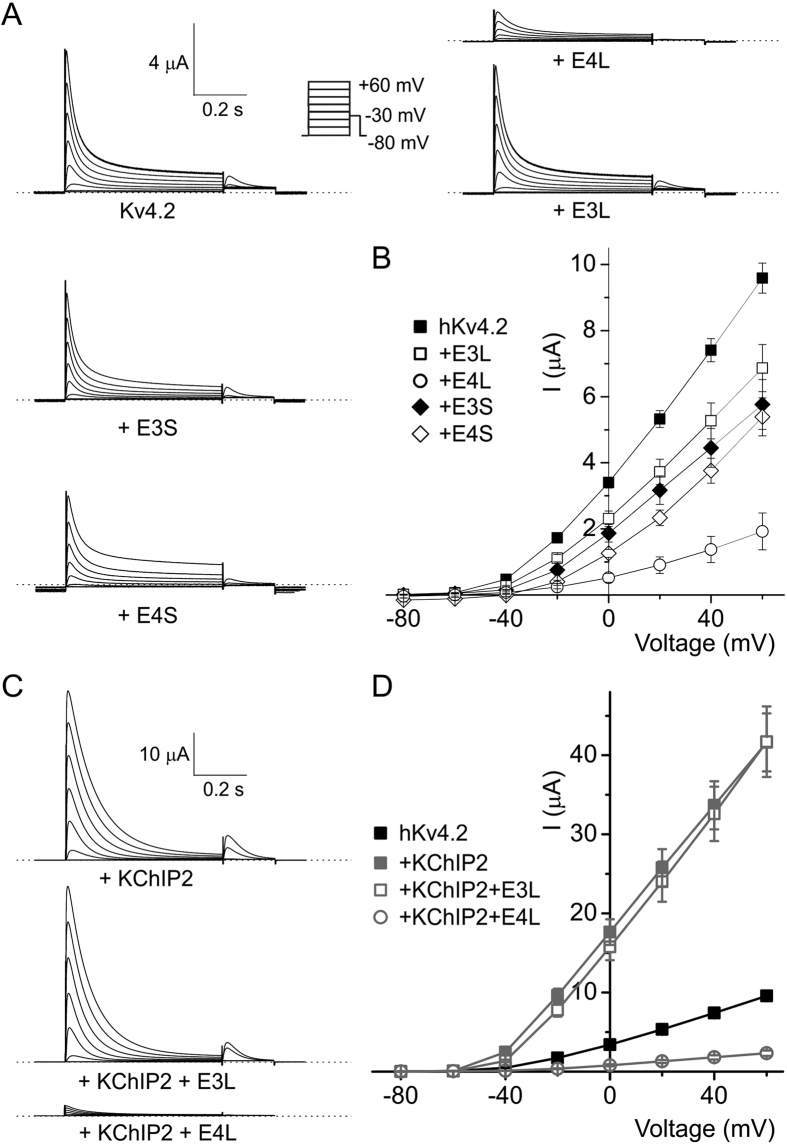
Effects of KCNE3L and hKCNE4L on human Kv4.2 current magnitude with/without KChIP2. (**A**) Exemplar current traces recorded from *Xenopus* oocytes expressing human Kv4.2 alone (*n* = 10) or with human KCNE3L (E3L; *n* = 9), KCNE4L (E4L; *n* = 10), KCNE3S (E3S; *n* = 12), or KCNE4S (E4S; *n* = 10). *Insets*: left, scale bars; right, Voltage clamp protocol. Zero current level indicated by dashed line. (**B**) Mean ± SEM peak current/voltage relationship for currents as in panel A; *n* values as in panel A. See main text for statistics. (**C**) Exemplar current traces recorded from *Xenopus* oocytes expressing human Kv4.2 + KChIP2 alone (*n* = 12) or with human KCNE3L (E3L; *n* = 12) or KCNE4L (E4L; *n* = 10). *Inset:* scale bars. Zero current level indicated by dashed line. (**D**) Mean ± SEM peak current/voltage relationship for currents as in panel C; *n* values as in panel C. See main text for statistics.

KCNE3S was previously reported to inhibit murine Kv4.2 in CHO cells^48^ and I recently recapitulated those findings using KCNE3S and rat Kv4.2 in oocytes^40^. Here, KCNE3S and KCNE3L each also moderately inhibited human Kv4.2 activity (30–40%; P < 0.005, *n* = 9–12) (Fig. 4A,B). In contrast, KCNE3L did not alter hKv4.2 current magnitude when co-expressed with KChIP2 (Fig. 4C,D). KCNE4S was previously found to have negligible effects on human Kv4.2 current density in tsA201 cells, with or without KChIP2^49^, whereas I previously found that KCNE4S inhibited rat Kv4.2 by ~40% in *Xenopus* oocytes, while KCNE4L had no effect on rat Kv4.2 current magnitude in oocytes (KChIP2 was not studied)^40^. Here, examining effects instead on human Kv4.2, KCNE4L inhibited its activity by 80% at +60 mV, while KCNE4S inhibited human Kv4.2 current by ~40% as it had done previously for rat Kv4.2 (Fig. 4A,B). Co-expression with hKChIP2 increased hKv4.2 currents fourfold, consistent with previous findings^50^, but did not protect from inhibition by KCNE4L, which was ~95% for Kv4.2-KChIP2 channels at +60 mV (Fig. 4C,D).

### Potent inhibition of hKv4.3 by KCNE4L is diminished by KChIP2

**Figure 5:**
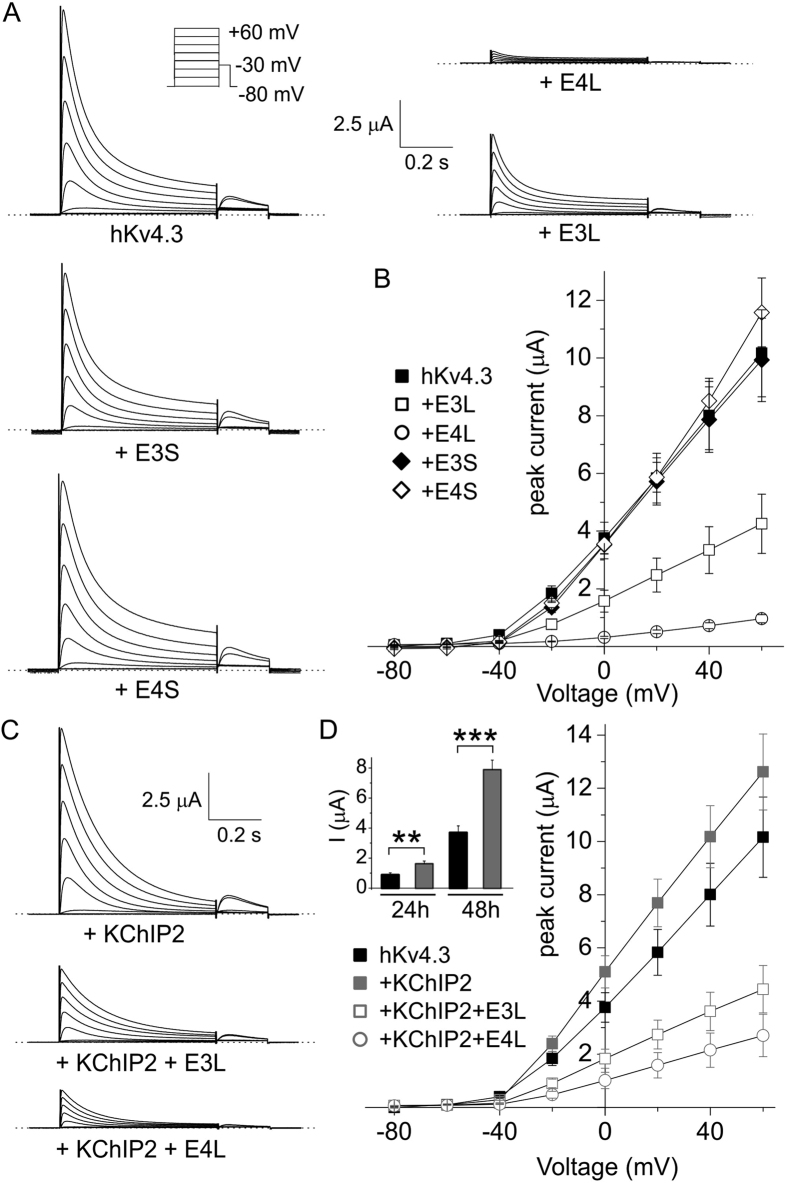
Effects of KCNE3L and hKCNE4L on human Kv4.3 current magnitude with/without KChIP2. (**A**) Exemplar current traces recorded from *Xenopus* oocytes expressing human Kv4.3 alone (*n* = 14) or with human KCNE3L (E3L; *n* = 13), KCNE4L (E4L; *n* = 13), KCNE3S (E3S; *n* = 11), or KCNE4S (E4S; *n* = 9) (5 ng cRNA per oocyte of each subunit injected) *Insets*: left, scale bars; right, Voltage clamp protocol. Zero current level indicated by dashed line. (**B**) Mean ± SEM peak current/voltage relationship for currents as in panel A; *n* values as in panel A. See main text for statistics. (**C**) Exemplar current traces recorded from *Xenopus* oocytes expressing human Kv4.3 + KChIP2 alone (*n* = 12) or with human KCNE3L (E3L; *n* = 12) or KCNE4L (E4L; *n* = 11) (5 ng cRNA per oocyte of each subunit injected). *Inset:* scale bars. Zero current level indicated by dashed line. (**D**) Mean ± SEM peak current/voltage relationship for currents as in panel C; *n* values as in panel C. See main text for statistics. *Inset*, Kv4.3 current augmentation at 24 h and 48 h expression when 1 ng Kv4.3 cRNA was co-injected with 1 ng KChIP2 cRNA (*n* = 10–13). **p < 0.01; ***p < 0.001.

KCNE3L and KCNE4L were each inhibitors of hKv4.3 activity, with the latter producing the strongest inhibition (~60% versus >90% at +60 mV, P < 0.005; *n* = 13–14). This was in contrast to KCNE3S and KCNE4S, neither of which altered Kv4.3 peak current (*n* = 9–11) (Fig. 5A,B). As previously reported^51^, KChIP2 doubled the peak whole-cell current at +40 mV of Kv4.3 (when 1 ng of each cRNA was co-injected per oocyte; Fig. 5D inset). Augmentation by KChIP2 was not apparent when a higher amount (5 ng per oocyte) of Kv4.3 cRNA was injected (P = 0.25, *n* = 12–14), suggesting that Kv4.3 membrane expression was already maximal after 5 ng cRNA injection, even without KChIP2 (Fig. 5C,D). Under these conditions, KChIP2 co-expression did not alter the extent of inhibition of hKv4.3 by KCNE3L (P = 0.89, *n* = 12) but was still able to partially alleviate inhibition by KCNE4L (from >90% to ~80% at +60 mV; P < 0.05, *n* = 11–12) (Fig. 5C,D).

### KCNE3L and KCNE4L differentially influence hKv4.x inactivation gating

**Figure 6:**
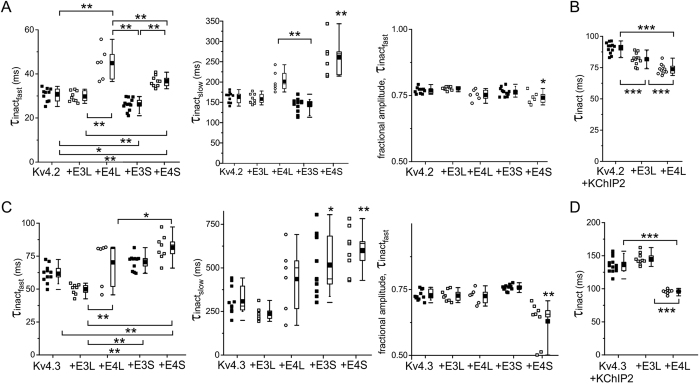
Effects of KCNE3L and hKCNE4L on human Kv4.2/3 inactivation kinetics with/without KChIP2. (**A**) Box plots showing individual and mean ± SEM values for τ of fast and slow components, and relative amplitude, of fast inactivation (double exponential fit) of Kv4.2 for currents recorded as in Fig. 4A; *n* = 6–12. *P < 0.05; **P < 0.01; by ANOVA followed by Tukey’s HSD test. Absence of brackets indicates P value versus all other groups. (**B**) Box plots showing individual and mean ± SEM values for τ of Kv4.2-KChIP2 inactivation (single exponential fit) for currents recorded as in Fig. 4C; *n* = 10–12. ***P < 0.0005. (**C**) Box plots showing individual and mean ± SEM values for τ of fast and slow components, and relative amplitude, of fast inactivation (double exponential fit) of Kv4.3 for currents recorded as in Fig. 5A; *n* = 6–11. *P < 0.05; **P < 0.01; by ANOVA followed by Tukey’s HSD test. Absence of brackets indicates P value versus all other groups. (**D**) Box plots showing individual and mean ± SEM values for τ of Kv4.3-KChIP2 inactivation (single exponential fit) for currents recorded as in Fig. 5C; *n* = 11–12. ***P < 1 × 10^−7^.

Fast inactivation is a signature of K^+^ channels formed by Kv4.x α subunits and is crucial to their role in shaping early action potential morphology in cardiac myocytes in a range of species including *Homo sapiens*. Here, inactivation at +40 mV of channels formed by Kv4.2 or Kv4.3 in the absence of KChIP2 was best fit with a double exponential function, while addition of KChIP2 produced inactivation that was well fit to a single exponential function. KCNE4L increased the τ of both the fast (by 50%) and slow (by 27%) components of hKv4.2 inactivation, and also slightly decreased the fractional amplitude of the fast component. KCNE4S exerted qualitatively similar effects but with quantitative differences that achieved statistical significance using ANOVA with post-hoc Tukey’s HSD to correct for multiple subunit combinations (Fig. 6A). KCNE3L did not alter hKv4.2 inactivation rate, while KCNE3S slightly speeded the fast component of inactivation (Fig. 6A). In contrast, hKv4.2-KChIP2 channel inactivation was speeded by both KCNE3L (~10%) and KCNE4L (~20%) (Fig. 6B).

KCNE3L decreased the τ of both the fast (20% at +40 mV) and slow (23% at +40 mV) components of hKv4.3 inactivation; this was opposite to the effects of KCNE3S and KCNE4S, which each slowed both components of hKv4.3 inactivation (Fig. 6C). KCNE4L appeared to induce a bimodal spread of hKv4.3 inactivation rates that was not statistically significantly different from those of Kv4.3 alone but could hint at an additional regulatory component being imposed by KCNE4L (Fig. 6C). In direct contrast, KCNE3L did not alter Kv4.3-KChIP2 inactivation rate, yet KCNE4L decreased its τ of inactivation 30% (P < 1 × 10^−7^, *n* = 8–13), qualitatively similar to previous reports for KCNE4S^52^.

**Figure 7:**
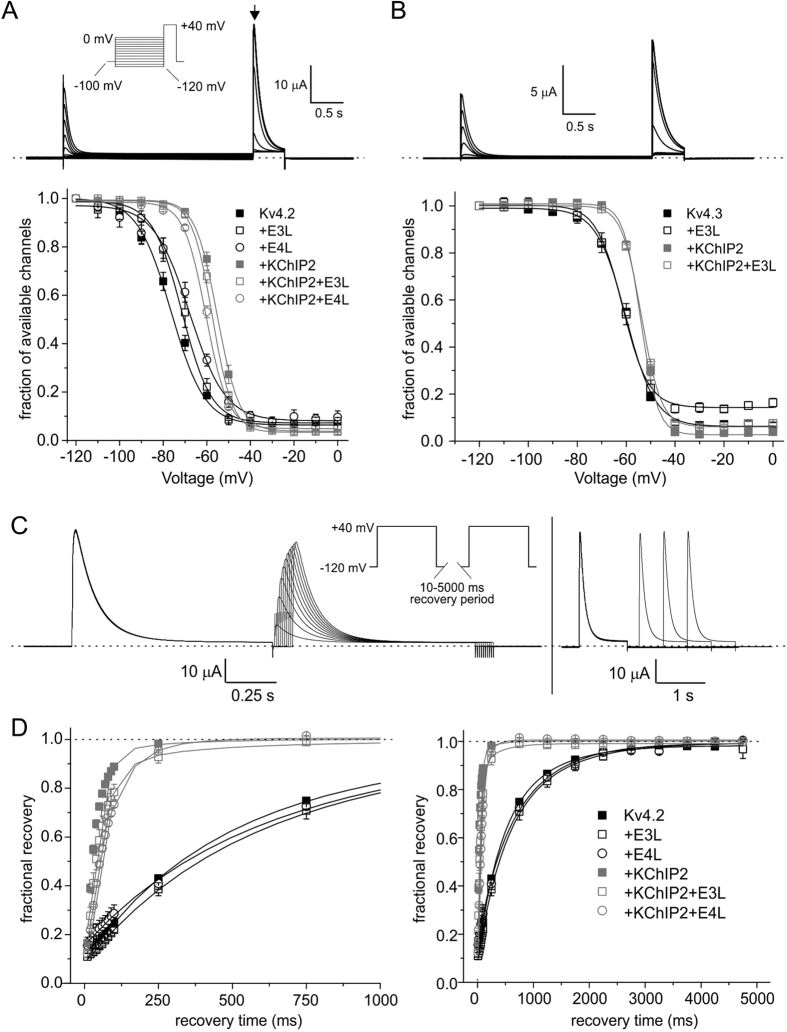
Effects of hKCNE3L and hKCNE4L on hKv4.2/3 steady-state inactivation and inactivation recovery with/without KChIP2. (**A**) *Upper*, exemplar current trace recorded from *Xenopus* oocyte expressing hKv4.2+ KChIP2, using steady-state inactivation protocol (*left inset*). Tail current time-point used for quantifying available channels indicated by arrow. Dashed line indicates zero current level. *Lower*, mean ± SEM values for voltage dependence of steady-state inactivation (plotted as fraction of channels available at arrow in panel A versus voltage) quantified using Voltage protocol as in panel A, for currents generated in *Xenopus* oocytes expressing hKv4.2 alone (*n* = 6) or with hKCNE3L (E3L; *n* = 4) or hKCNE4L (E4L; *n* = 6); or Kv4.2+ KChIP2 alone (*n* = 6), or with hKCNE3L (E3L; *n* = 5) or hKCNE4L (E4L; *n* = 6). (**B**) *Upper*, exemplar current trace recorded from *Xenopus* oocyte expressing hKv4.3+ KChIP2, using steady-state inactivation protocol as in panel A. Tail current time-point used for quantifying available channels indicated by arrow. Dashed line indicates zero current level. *Lower*, mean ± SEM values for voltage dependence of steady-state inactivation (fraction of channels available at arrow in panel A versus voltage) quantified using Voltage protocol as in panel A, for currents generated in *Xenopus* oocytes expressing hKv4.3 alone (*n* = 6) or with hKCNE3L (E3L; *n* = 5); or hKv4.3 + KChIP2 alone (*n* = 9), or with hKCNE3L (E3L; *n* = 7). (**C**) *Upper*, exemplar current traces recorded using “short” and “long” inactivation recovery protocols (inset) from a *Xenopus* oocyte expressing hKv4.2 + KChIP2. Dashed line indicates zero current level. (**D**) Mean ± SEM values for the time course of recovery from inactivation for currents generated as in panel C in *Xenopus* oocytes expressing hKv4.2 alone (*n* = 6) or with hKCNE3L (E3L; *n* = 5) or hKCNE4L (E4L; *n* = 6); or Kv4.2 + KChIP2 alone (*n* = 6), or with hKCNE3L (E3L; *n* = 6) or hKCNE4L (E4L; *n* = 6). *Left*, close-up of first 1s of recovery; *right*, full 5s recovery period.

KCNE3L and KCNE4L each shifted the voltage dependence of Kv4.2 steady-state inactivation more positive, from a V_1/2_ of −76 ± 0.7 mV (Kv4.2 alone) to −71.4 ± 1 mV (with KCNE3L) and −67.9 ± 1 mV (KCNE4L). KChIP2 positively shifted Kv4.2 steady-state inactivation V_1/2_ by >20 mV, to −55.1 ± 0.5 mV (similar to previous reports^53^); this shift was slightly attenuated, by +2 mV for KCNE3L and +5 mV for KCNE4L. KChIP2 altered the slope from ~8 mV to ~5 mV but neither KCNE subunit impacted this property (Fig. 7A). KChIP2 also positive-shifted and steepened the slope of voltage dependence of Kv4.3 steady-state inactivation, while KCNE3L had no effects on either property for Kv4.3 or Kv4.3-KChIP2 channels (Fig. 7B). Increases in the fractional current remaining at −50 mV to 0 mV associated with KCNE3L co-expression with Kv4.3 probably arose from increased relative contribution of endogenous current because of Kv4.3 inhibition by KCNE3L (Fig. 7B). For subunit combinations in which KCNE4L exerted strong inhibitory effects, steady-state inactivation could not be meaningfully quantified and these were omitted.

hKv4.2 recovery from inactivation fitted well with a double exponential fit regardless of the subunits regulating it. In the absence of KChIP2, recovery from inactivation of hKv4.2 was slow and unaffected by KCNE3L or KCNE4L (Fig. 7C,D). Differences in the amplitudes of fast and slow components of hKv4.2-KCNE4L recovery compared to those of hKv4.2 alone likely arise from difficulties in fitting the much smaller currents after KCNE4L inhibition and correspondingly increased relative contribution of endogenous current (Table 1). In contrast, KCNE3L and KCNE4L each slowed recovery from inactivation of hKv4.2-KChIP2 channels, which in the absence of KCNEs is much faster than that of hKv4.2^50^, both inducing increases in the τ of fast inactivation and a reduction in its relative amplitude. Consequently, at least double the fraction of channels remained inactivated after 100 ms of recovery at −120 mV in hKv4.2-KChIP2 channels co-expressed with KCNE3L (P = 0.01) or KCNE4L (P = 1 × 10^−6^), compared to those without (*n* = 6 per group) (Fig. 7C,D; Table 1). Interestingly, KCNE4L produced a minor overshoot in hKv4.2-KChIP2 channels (Fig. 7D). A complete comparison of effects of KCNE3L and KCNE4L on recovery from inactivation of hKv4.3 channels was not feasible because current inhibition for some subunit combinations precluded accurate fitting.

### Conclusions, limitations and future studies

The current work expands our understanding of the potential modes of regulation of human cardiac potassium channels by demonstrating the effects of newly discovered longer forms of human KCNE3 and KCNE4 regulatory subunits on human channel properties. While some features of KCNE3L and KCNE4L function are shared with their shorter counterparts, other properties differ. Most markedly, KCNE4L is a potent inhibitor of Kv4.3 activity, even in the presence of the cytosolic regulatory subunit, KChIP2. This unexpected finding raises the possibility that KCNE4L could act as a repressor subunit for *I*_to_, the transient outward K^+^ current thought to be primarily generated by Kv4.3-KChIP2 complexes in human cardiac myocytes. This might contribute to transmural gradients of *I*_to_ across the myocardium, or perhaps allow dynamic regulation of *I*_to_ density and other properties in response to specific signals or during different developmental states.

The recent discovery of extended forms of human KCNE3 and KCNE4 proteins opens up the potential for another level of complexity with regard to regulation of potassium channels by the KCNE subunits. Specifically, it may be possible for cells to respond to different signals by expressing either long or short forms of KCNE3 and KCNE4, with different functional outcomes. However, elucidation of whether splice variation occurs, versus solely the long forms being transcribed, and the mechanisms regulating this, will require future studies. Similarly, the question still remains of exactly which complexes can form in native tissues including the heart, and how the subunit composition might vary between cells or even within a single cell. Partly because KCNE proteins are small, transmembrane subunits, and partly because K^+^ channels need not necessarily be expressed at high levels in excitable cells, cataloging of precise channel subunit composition at a regional level is highly challenging. The discovery of new, extended forms of KCNE3 and KCNE4 further complicates attempts at these assignments.

Sequence variations in the human KCNE3 gene have been discovered and tentatively functionally associated with propensity to cardiac arrhythmias including LQTS^54^, Brugada syndrome^34^,^36^ and atrial fibrillation^55^. In addition, a single polymorphism in the human KCNE4 gene (E145D) is suggested to alter predisposition to atrial fibrillation in Chinese populations^56^,^57^,^58^,^59^. In future studies it will be of interest to determine whether the presence of the additional N-terminal residues in KCNE3L or KCNE4L, versus their shorter counterparts, alters the functional consequences of the disease-associated gene sequence variants.

## Additional Information

**How to cite this article**: Abbott, G. W. Regulation of human cardiac potassium channels by full-length KCNE3 and KCNE4. *Sci. Rep.* **6**, 38412; doi: 10.1038/srep38412 (2016).

**Publisher's note:** Springer Nature remains neutral with regard to jurisdictional claims in published maps and institutional affiliations.

## Figures and Tables

**Table 1:** Effects of KCNE3L, KCNE4L and KChIP2 on Kv4.2 Inactivation recovery kinetics.

Subunits	τ_fast_ (ms)	A_fast_	τ_slow_ (ms)	A_slow_	Chi^2^
Kv4.2	344 ± 50	0.41 ± 0.13	809 ± 104	0.48 ± 0.13	<0.005
Kv4.2 + E3L	423 ± 200	0.43 ± 0.46	978 ± 450	0.48 ± 0.46	<0.005
Kv4.2 + E4L	309 ± 123	0.16 ± 0.06	fixed at 800	0.67 ± 0.07	<0.005
Kv4.2-KChIP2	37 ± 3.1	0.94 ± 0.07	248 ± 444	0.06 ± 0.07	<0.005
Kv4.2-KChIP2 + E3L	50 ± 3.2	0.89 ± 0.05	345 ± 232	0.1 ± 0.05	<0.005
Kv4.2-KChIP2 + E4L	65.9 ± 13.7	0.87 ± 0.39	152 ± 168	0.16 ± 0.01	<0.005

Fits were made to mean data and therefore P values are not available for comparisons between groups, but Chi^2^ values are given to indicate goodness of fit. For graphs of fits see Fig. 7.
